# Anthocyanin Degrading and Chlorophyll Accumulation Lead to the Formation of Bicolor Leaf in Ornamental Kale

**DOI:** 10.3390/ijms20030603

**Published:** 2019-01-30

**Authors:** Jie Ren, Zhiyong Liu, Weishu Chen, Hezi Xu, Hui Feng

**Affiliations:** Department of Horticulture, Shenyang Agricultural University, 120 Dongling Road, Shenhe District, Shenyang 110866, China; m18240088920@163.com (J.R.); lzyky99@163.com (Z.L.); cws.005@163.com (W.C.); 15809881913@163.com (H.X.)

**Keywords:** *Brassica oleracea* L. var. *acephala*, anthocyanin, chlorophyll, transcriptome

## Abstract

Ornamental kale is a popular decorative plant. We identified a peculiar bicolor leaf double haploid line, with green margins and red centers. The development of bicolor leaves can be divided into three stages: S1, S2, and S3. To probe the reason for bicolor formation, we analyzed the anthocyanin and chlorophyll contents, detected the changes in indole-3-acetic acid (IAA), abscisic acid (ABA), gibberellin 3 (GA3), sugar, and starch contents, and identified the differentially expressed genes (DEGs) using RNA-seq. Results showed that the bicolor leaf phenotype is gradually formed with anthocyanin degrading and chlorophyll accumulation. Anthocyanin content is lower in the green margin (S3_S) than in the red center (S3_C) part at S3. IAA content was positively correlated with anthocyanin content during the bicolor leaf development. During anthocyanin degrading from S1 to S2, *cinnamate-4-hydroxylase* (*C4H*) and *transport inhibitor response 1* (*TIR1*) were downregulated, while *lateral organ boundaries domain 39* (*LBD39*) was upregulated. Two peroxidases, two β-glucosidases (*BGLU*), *LBD39*, *LBD37*, *detoxifying efflux carrier 35* (*DTX35*), three *no apical meristem* (*NAC*) transcription factors (TFs), and 15 WRKY DNA-binding protein (WRKY) TFs were downregulated in S3_S *vs.* S3_C. The bicolor phenotype was mainly linked to anthocyanin degrading and chlorophyll accumulation, and that anthocyanin degrading resulted from reduced anthocyanin biosynthesis and increased anthocyanin degradation.

## 1. Introduction

**Figure 1:**
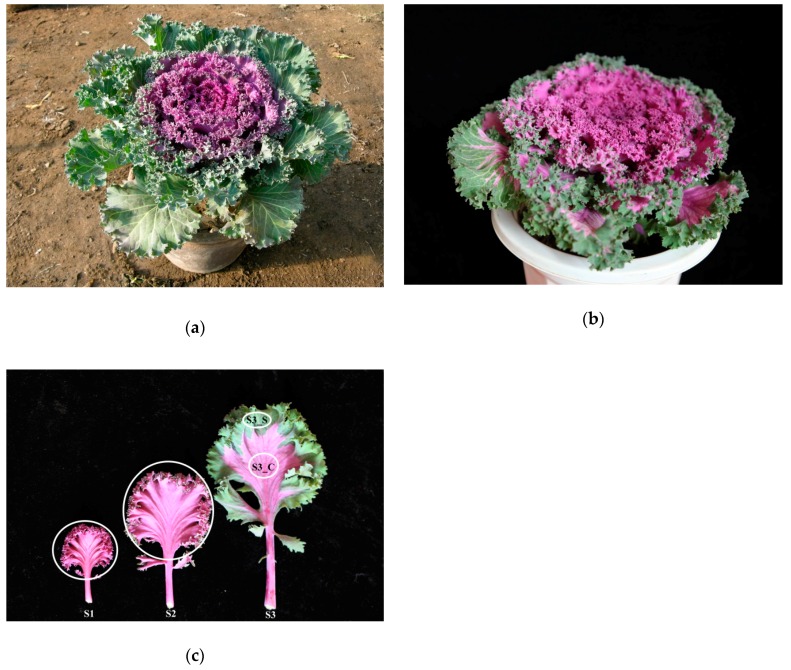
Phenotype of ‘Y005-7’ and the progenitor. (**a**) Phenotype of the progenitor. (**b**) Phenotype of ‘Y005-7’. (**c**) Phenotype of ‘Y005-7’ at the different developmental stages. The green margin at S3 was denoted as S3_S, and the red center was denoted as S3_C. The white circles denote the sampling sites for RNA extraction, pigment, hormone, sugar, and starch content measurement.

Ornamental kale (*Brassica oleracea L.* var. *acephala*) is popular because of its bright color and various leaf shapes. Most ornamental plants bear flowers with various colors and pigmentation patterns, spots, irregular blotches, venation, and others [1]. In other plant species, variegated, striped, transparent, yellow, and spotted leaves are attractive features [2]. Using an isolated microspore culture from the Kamome Red variety (Figure 1a,b), we identified a peculiar bicolor leaf, double haploid (DH) line, Y005-7, of ornamental kale, showing green margins around a red center. The S3 leaf of Y005-7 showed a bicolor leaf, while the S3 leaf of the Kamome Red variety was green. This is a novel ornamental trait with great commercial potential. Thus, to elucidate the mechanism of leaf color formation, it is necessary to investigate the pigment change process.

Anthocyanin is a water-soluble pigment. In leaves, it imparts red, pink, blue, purple, and other colors [3]. The anthocyanin biosynthetic pathway is a secondary metabolic process and it has been the subject of extensive study [4,5,6]. This pathway is divided into four stages: the phenylpropanoid pathway stage, polyketide pathway stage, the early biosynthesis stage, and the later biosynthesis stage. The phenylpropanoid pathway generates p-coumaroyl-coenzyme A (CoA) as substrates for anthocyanin biosynthesis. Chalcone synthase (CHS), chalcone isomerase (CHI), flavanone 3-hydroxylase (F3H), and flavonoid 3′-hydroxylase (F3′H) participate in early biosynthesis. The polyketide pathway provides malonyl-CoA for anthocyanin biosynthesis. Flavonol synthase forms flavonol kaempferol. Dihydroflavonol reductase (DFR), leucoanthocyanidin dioxygenase/anthocyanidin synthase (LDOX/ANS), and anthocyanidin reductase (ANR) catalyze the formation of anthocyanin and proanthocyanin [7]. After glycosylation, methylation, and acylation, these compounds are transported to the vacuoles by glutathione-S-transferase (GST), the adenosine triphosphate (ATP)-binding cassette (ABC), and toxic compound extrusion (MATE) Family proteins [8].

The genes involved in the regulation of anthocyanin biosynthesis have also been identified. Both positive and negative regulators of anthocyanin biosynthesis have been described. Members of the R2R3-myeloblastosis (MYB) gene family, basic helix-loop-helix transcription factors, and WD40 repeat proteins form a complex (MBW) that activates the expression level of the structure genes. This complex also participates in other pathways, seed mucilage formation and epidermal cell fate determination, among others [9]. Transcription factor HY5 homolog (HYH) and elongated hypocptyl 5 (HY5) family genes regulate *CHI*, *CHS*, *F3H*, and *DFR* expressions at low temperatures [10]. Two single-repeat R3-MYB transcription factors (MYB-like 2 (MYBL2) and caprice (CPC)), three members of the lateral organ boundaries domain (LBD) family (LBD37, LBD38, and LBD39), and squamosa promoter binding protein-like 9 (SLP9) negatively regulate anthocyanin biosynthesis in *Arabidopsis thaliana* [11,12,13,14]. Genes *LBD37*, *LBD38*, and *LBD39* negatively regulate the late anthocyanin-specific steps by repressing the production of the anthocyanin pigment (PAP) genes PAP1 and PAP2 under nitrogen/nitrate induction. Recently, other TFs, WRKY and NAC, were also found to regulate anthocyanin biosynthesis [15,16].

In addition to endogenous genes, other factors play an important role in anthocyanin accumulation. For example, MBW decreased under low light and high temperature; thus, further influencing anthocyanin content [17]. Exogenous sugar treatment could increase *CHS*, *CHI*, *F3H*, *F3*′*H*, *DFR*, and *LDOX* expression [18]. Lewis et al. (2011) reported that IAA plays a positive role in anthocyanin biosynthesis; cytokinin (CK), ABA, and GA also promote anthocyanin biosynthesis [19,20]. 

Recently, the anthocyanin degradation process in plants has been revealed and this metabolic process has received more attention. Anthocyanin usually accumulates in young leaves, and degrades in mature leaves. In young leaves, anthocyanin protects the cells from ultraviolet (UV) light damage, especially in photosynthesis apparatus. As the leaves mature, the leaf color often changes from red to green. Some enzymes have also been reported as involved in anthocyanin degradation in fruit, juice, and flower. For example, β-glucosidases remove the sugar moieties, while the class III peroxidases oxidize the aglycone [21]. Fang et al. (2015) identified a laccase (ADE/LAC) responsible for anthocyanin degradation in litchi fruit pericarp [22]. However, few studies have focused on anthocyanin degradation in plant leaves, which is the subject of the present study.

Chlorophyll is a lipid-soluble pigment located in the thylakoid membrane. It plays a core role in light absorption for photosynthesis. It also absorbs most red and purple wavelengths, but reflects green. The chlorophyll metabolic pathway in higher plants is well characterized. It consists of three steps: chlorophyll biosynthesis, chlorophyll cycle (interconversion of chlorophyll a and chlorophyll b), and chlorophyll degradation [23,24].

In the present study, we obtained a bicolor leaf mutant ‘Y005-7’ (Figure 1a) and analyzed its physiological traits. Total RNA was sampled from the whole leaf without the main vein (stages 1 and 2), and from the margin and center sectors (stage 3) of ‘Y005-7’ and subject to deep sequencing to obtain gene expression profiles related with anthocyanin biosynthesis, chlorophyll biosynthesis, and anthocyanin degradation. A set of DEGs were also identified as involved in anthocyanin degrading and bicolor formation. This information might be applied for breeding plants with desirable color traits and it lays the foundation for further genetic studies on anthocyanin degradation in ornamental kale and other plants.

## 2. Results

### 2.1. Chlorophyll and Anthocyanin Levels in Leaves

**Figure 2:**
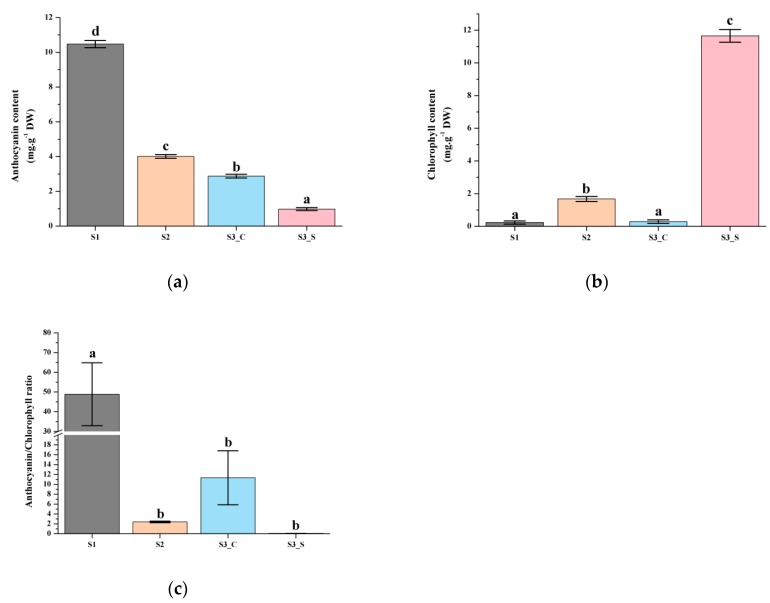
Pigment accumulation in ‘Y005-7’ leaves at different developmental stages. (**a**) Anthocyanin contents at S1–S3. (**b**) Chlorophyll contents at S1–S3. (**c**) Anthocyanin/chlorophyll ratio at S1–S3. Different letters among stages indicate significant differences at *p* ≤ 0.01 based on the analysis of variance (ANOVA) (Tukey test).

Anthocyanin content was highest at S1 and decreased at S2, as it reached 10.47 mg g^−1^ DW (dry weight) at S1 before subsequently declining to 4 mg g^−1^ DW at S2 (Figure 2a). The chlorophyll contents were very low at S1 and S2 (Figure 2b). The leaf matured and at S3 it finally exhibit green margin and red center. Low levels of anthocyanin were detected in green sectors at S3 (Figure 2a). Chlorophyll levels in the center and margins were also significantly different; the central part contained much less chlorophyll, while the margins contained high chlorophyll levels (Figure 2b). We also compared the anthocyanin/chlorophyll ratio and found that it ranged from 0.08 to 48.89. The ratio was highest at S1 and lowest at S3_S; the value at S2 was 2.40, and chlorophyll started to accumulate when it was below 2.40 (Figure 2c).

Based on these results, we hypothesized that increased chlorophyll biosynthesis and anthocyanin degrading lead to the bicolor leaf formation. According to the measurement results of these pigments, we divided the development into two parts to evaluate anthocyanin degrading from S1 to S2 and the different anthocyanin degrading and chlorophyll accumulation rate at S3.

### 2.2. IAA, ABA, GA3, Sugar, and Starch Contents in Leaves

Previous studies showed that plant hormone and sugar contents are related with anthocyanin accumulation. To further confirm whether hormones played an important role in anthocyanin content changes, IAA, ABA and GA3 contents were examined. Total sugar and starch contents were also measured to test whether hormone-regulated anthocyanin content changes depend on sugar and starch changes, and if sugar and starch independently regulate anthocyanin content changes.

**Figure 3:**
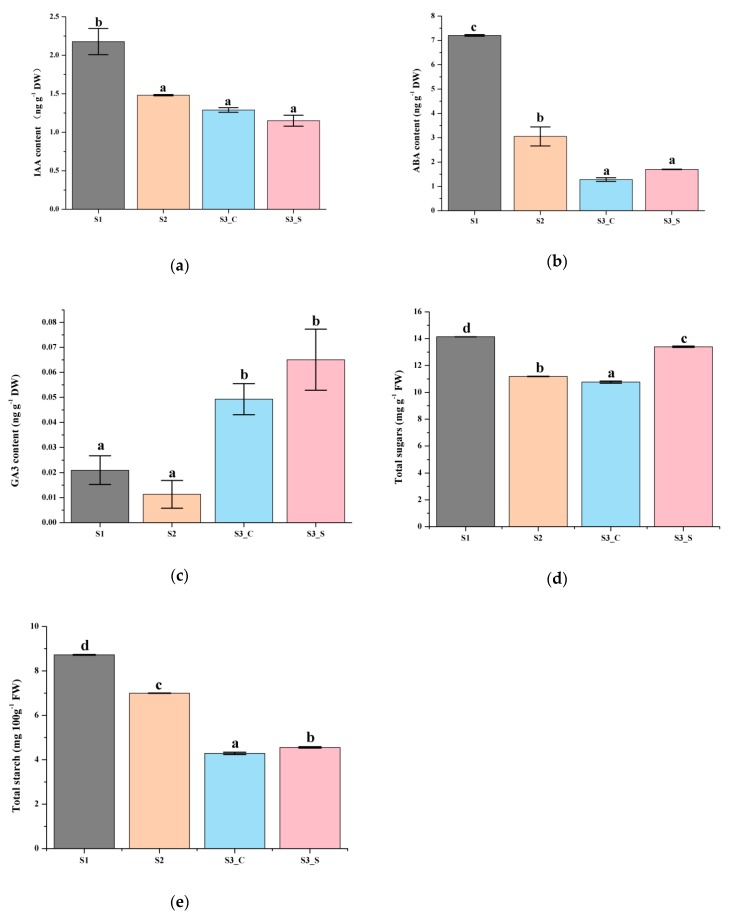
Biochemical analysis of leaves at different developmental stages. (**a**) IAA content at S1-S3. (**b**) ABA content at S1–S3. (**c**) GA3 content at S1–S3. (**d**) Total sugar content at S1–S3. (**e**) Starch content at S1–S3. Different letters among stages indicate significant differences at *p* ≤ 0.05 based on ANOVA (Tukey test).

The red leaves in S1 contained higher IAA, ABA and GA3 contents than that in S2. ABA and GA3 contents were higher in the green margins of S3 leaves (S3_S) than in the red centers (S3_C), while the IAA content showed the opposite pattern (Figure 3a–c). Moreover, the GA3 contents in the center and margin of leaves at S3 were higher than that in S1 and S2 leaves. Only the changes in IAA content in different parts of the leaf and at the different stages studied were similar to changes in anthocyanin content, suggesting that IAA was positively correlated with anthocyanin biosynthesis and degradation. Thus, IAA might play an important role in anthocyanin content changes regardless of changes in sugar and starch contents.

### 2.3. Illumina Sequencing, Sequence Assembly, and DEG Analyses

**Figure 4:**
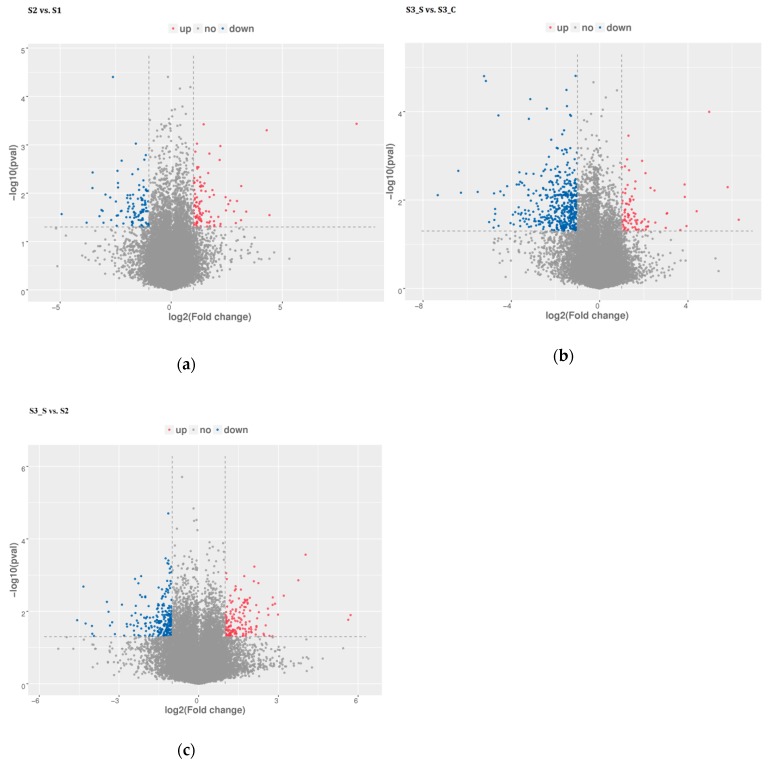
Distribution of differential expressed genes in pairwise comparisons. (**a**) Differential expressed genes in S2 *vs.* S1. (**b**) Differential expressed genes in S3_S *vs.* S3_C. (**c**) Differentially expressed genes in S3_S *vs.* S2.

Pairwise comparisons were conducted to identify the candidate genes involved in anthocyanin biosynthesis and degradation at S2 and in the formation of bicolor leaves at S3. Of the 588.64 million raw reads obtained, 582.72 million were classified as clean after filtering out the low-quality reads (Table S1). Pairwise comparisons revealed 201, 543, and 391 DEGs in S2 *vs.* S1, S3_S *vs.* S3_C and S3_S *vs.* S2, respectively (Figure 4a–c, Tables S2–S4). The pairwise comparison of S2 *vs.* S1 revealed 99 upregulated and 102 downregulated DEGs, while that of S3_S *vs.* S3_C revealed 87 upregulated and 456 downregulated DEGs, and that of S3_S *vs.* S2 revealed 152 upregulated and 239 downregulated DEGs. Identified DEGs included genes involved in anthocyanin biosynthesis, anthocyanin degradation, and photosynthesis. DEGs identified in the pairwise comparisons of S2 *vs.* S1, S3_S *vs.* S3_C, and S3_S *vs.* S2, were selected as the candidate genes potentially associated with anthocyanin degradation and formation of bicolor leaves in ornamental kale and were subjected to further functional analysis.

### 2.4. Functional Annotation and Classification

**Figure 5:**
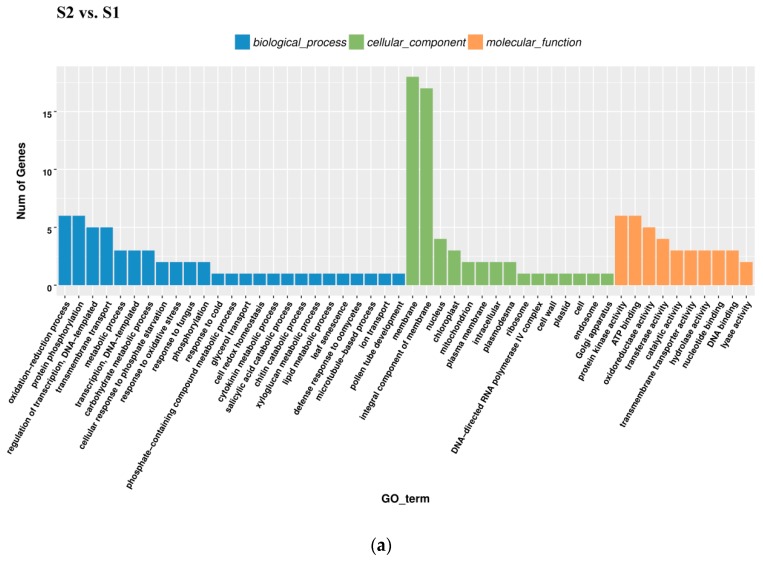

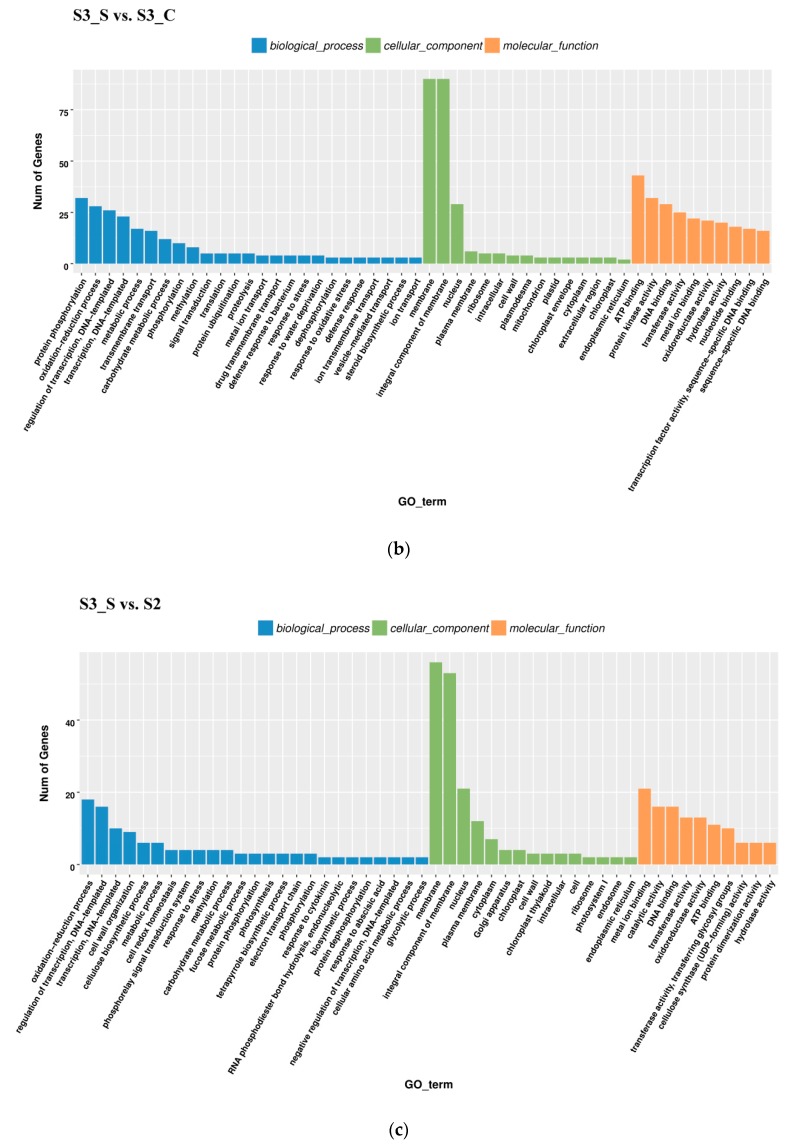
Gene Ontology (GO) enrichment analysis in pairwise comparisons. (**a**) GO enrichment analysis in S2 *vs.* S1. (**b**) GO enrichment analysis in S3_S *vs.* S3_C. (**c**) GO enrichment analysis in S3_S *vs.* S2.

All DEGs identified in pairwise comparisons were searched in the GO (Gene Ontology) and KEGG (Kyoto Encyclopedia of Genes and Genomes) pathway databases. In the S2 *vs.* S1 pairwise comparison, DEGs were significantly enriched in 49 GO terms as follows: 29 terms for biological process, 18 terms for molecular function, and 1 term for cellular component. The oxidation-reduction process contained the largest number of genes, and therefore anthocyanin degrading might be related with the oxidation-reduction process (Figure 5a). Downregulated DEGs were enriched in 12 pathways, including anthocyanin related ‘Flavonoid biosynthesis’ and ‘Phenylpropanoid biosynthesis’.

**Figure 6:**
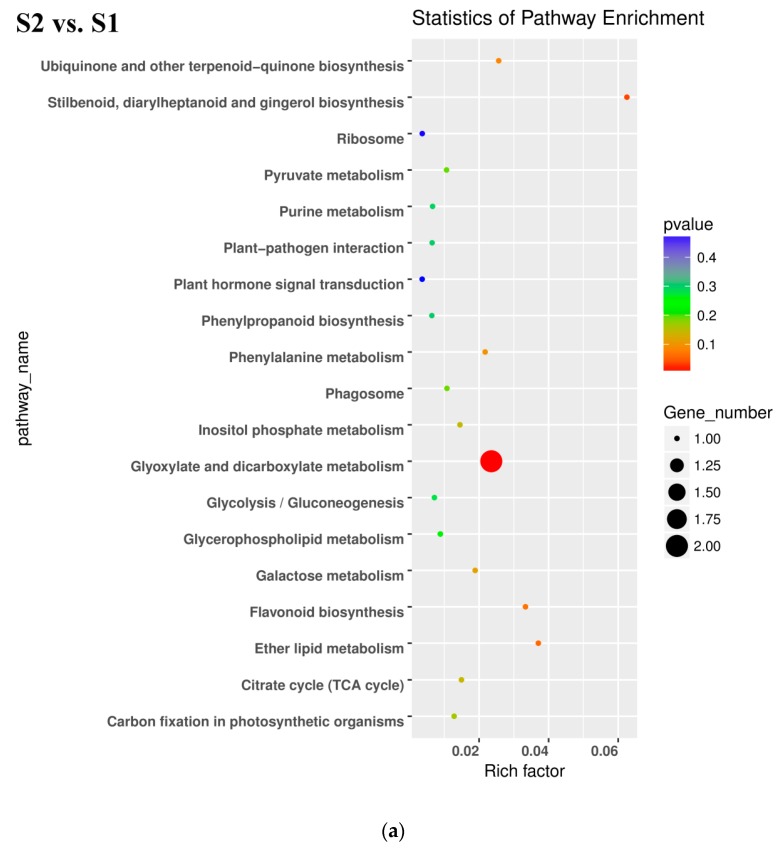

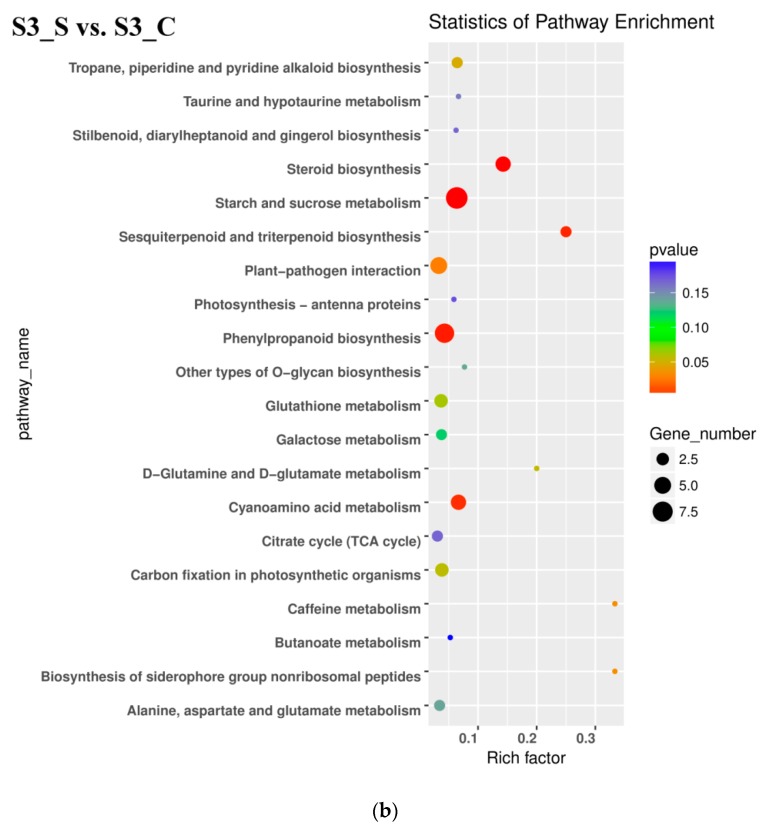

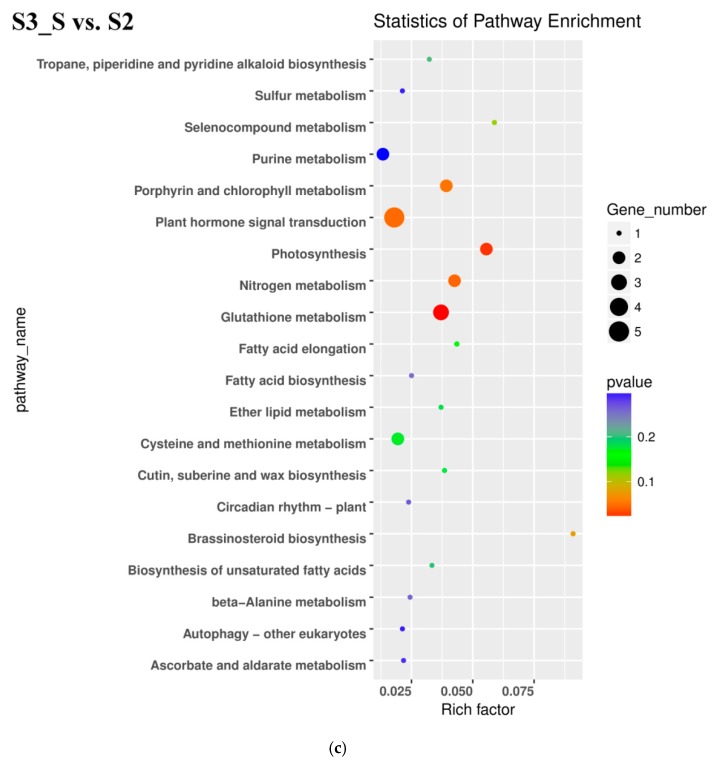
Kyoto Encyclopedia of Genes and Genomes (KEGG) enrichment analysis in pairwise comparisons. (**a**) KEGG enrichment analysis in S2 *vs.* S1. (**b**) KEGG enrichment analysis in S3_S *vs.* S3_C. (**c**) KEGG enrichment analysis in S3_S *vs.* S2.

In the S3_S *vs.* S3_C pairwise comparison, DEGs were significantly enriched in 83 GO terms as follows: 43 terms for biological process, 36 terms for molecular function, and 4 terms for cellular component. Protein phosphorylation included the largest number of genes related with biological process, while oxidation-reduction included the second (Figure 5b). KEGG enrichment analysis showed that the DEGs were significantly enriched in the pathway of ‘Starch and sucrose metabolism’, ‘Steroid biosynthesis’, ‘Phenylpropanoid biosynthesis’, ‘Sesquiterpenoid and triterpenoid biosynthesis’, ‘Cyanoamino acid metabolism’, ‘Plant-pathogen interaction’, ‘Biosynthesis of siderophore group nonribosomal peptides’, ‘Caffeine metabolism’, and ‘Tropane, piperidine, and pyridine alkaloid biosynthesis’ (Figure 6b). Downregulated DEGs were enriched in 43 pathways, including anthocyanin related ‘flavonoid biosynthesis’, ‘peroxisome’ and ‘phenylpropanoid biosynthesis’, while upregulated DEGs were enriched in 7 pathways, including ‘photosynthesis’. Phenylpropanoid biosynthesis is one part of anthocyanin biosynthesis, and changes in this pathway might be related with anthocyanin degrading and bicolor formation at S3.

In the pairwise comparison of S3_S *vs.* S2, DEGs were significantly enriched in 64 GO terms as follows: 32 terms for biological process, 25 terms for molecular function, and 7 terms for cellular component. Oxidation-reduction included the largest number of genes related with biological process (Figure 5c). KEGG enrichment analysis showed that DEGs were significantly enriched in the following pathways: ‘glutathione metabolism’, ‘photosynthesis’, ‘nitrogen metabolism’, ‘sesquiterpenoid and triterpenoid biosynthesis’ and ‘plant hormone signal transduction’ (Figure 6c). Up- and downregulated DEGs were distributed into in 18 pathways each. Upregulated DEGs were enriched in ‘photosynthesis’ and ‘porphyrin and chlorophyll metabolism,’ which might be related with chlorophyll accumulation at S3.

### 2.5. Functional Annotation and Classification

Because bicolor leaf formation was related with anthocyanin degrading and chlorophyll accumulation, and the anthocyanin biosynthesis and chlorophyll metabolism have been well studied, the protein query basic local alignment search tool (BLASTP) method and GO and KEGG analysis were used to identify the genes involved in anthocyanin and chlorophyll metabolism in ornamental kale. The KEGG analysis was used to identify the peroxidases (Enzyme (EC) 1.11.1.7) and the gene annotation was used to identify the β-glucosidases and laccases in anthocyanin degradation.

Ninety-eight genes were involved in anthocyanin biosynthesis in ornamental kale, including 52 structural genes encoding anthocyanin biosynthesis enzymes and the intermediate and final products of this pathway, 34 genes encoding special regulatory proteins, four genes encoding proteins transporting anthocyanin and proanthocyanin to the vacuoles, and eight other genes (Table S5). One-hundred and nineteen genes involved in chlorophyll metabolism and its regulation were also identified, including 71 genes involved in chlorophyll metabolism and 48 genes related with the regulation of chlorophyll metabolism (Table S6). Polyphenol oxidase is located in the chloroplast, while anthocyanin is synthesized in the cytoplasm and transported to the vacuole. Although the link between vacuole and chloroplast is still unknown, the polyphenol oxidase gene should be excluded from the candidate genes. Sixty peroxidases, 13 β-glucosidases, and 16 laccases were finally identified (Table S7).

### 2.6. Quantitative Real-Time PCR (qRT-PCR) Analysis of the Gene Expression Patterns 

**Figure 7:**
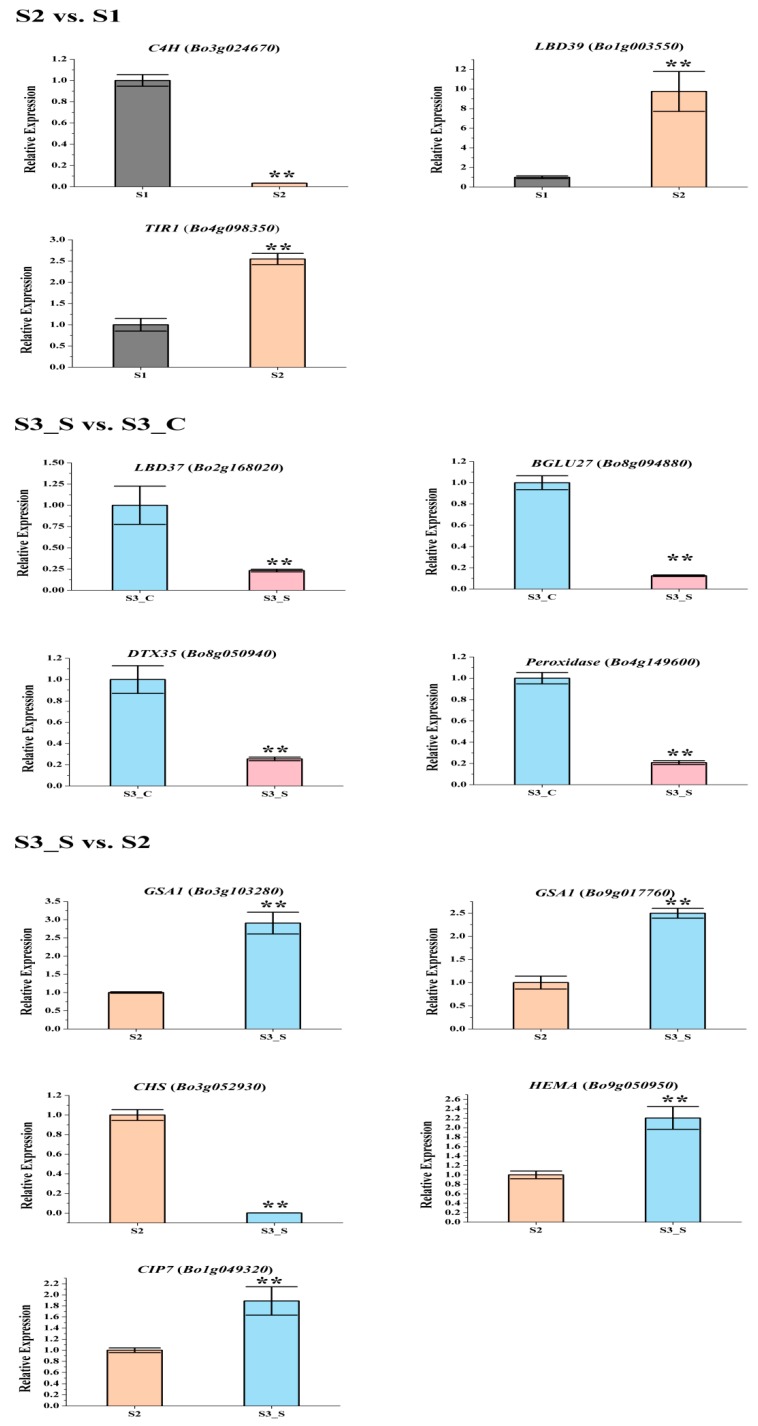
Expression levels of the differentially expressed genes (DEGs) related with anthocyanin biosynthesis, anthocyanin degrading and chlorophyll biosynthesis in the three pairwise comparisons. Only DEGs with reduction in S3_S *vs.* S3_C were tested. Data are represented as the mean ± standard deviation (SD). ** *p* < 0.01 using the t-test.

The gene expression patterns of the DEGs involved in pigment biosynthesis were validated by qRT-PCR (Figure 7, Table S8), which indicated that the transcriptome analysis was reliable.

### 2.7. Analysis of the Genes Involved in Anthocyanin Biosynthesis

**Figure 8:**
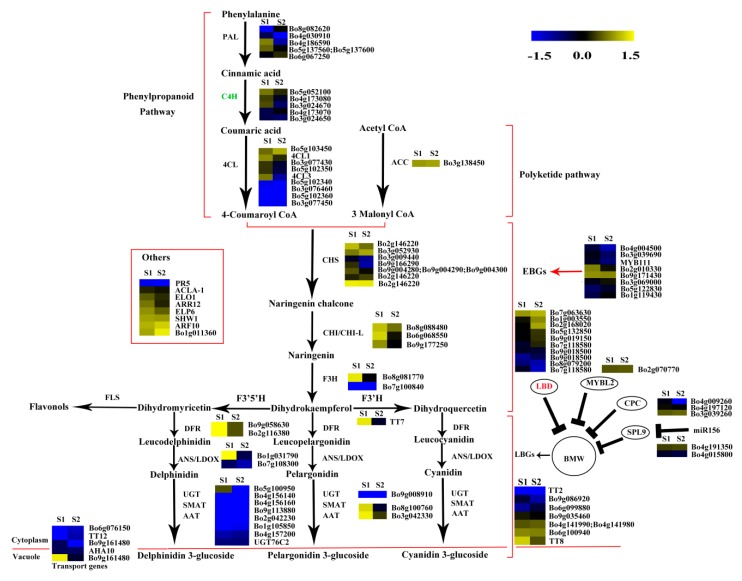
Expression patterns of the genes involved in anthocyanin biosynthesis in S2 *vs.* S1. Enzyme names in red represent upregulated genes in S2 *vs.* S1. Enzyme names in green represent downregulated genes in S2 *vs.* S1. The red parentheses represent the four pathways, the black and red arrows represent the positive regulation and black “T” represent negative regulation. The genes in the red box are the other genes involved in anthocyanin biosynthesis.

**Figure 9:**
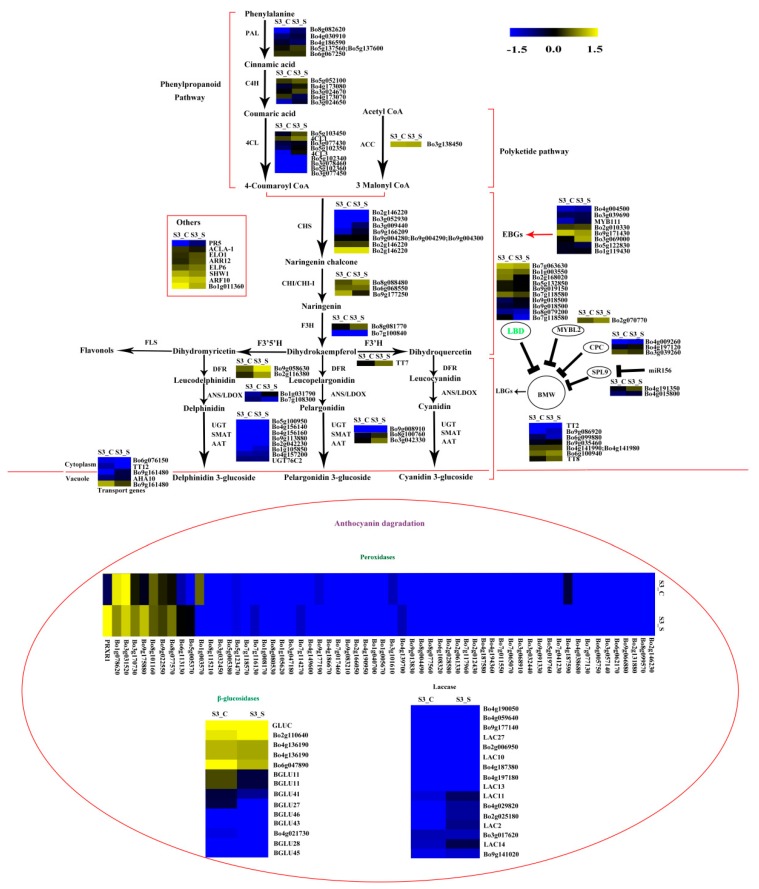
Expression pattern of the genes involved in anthocyanin biosynthesis and anthocyanin degradation in S3_S *vs.* S3_C. Enzyme names in green represent downregulated genes in S3_S *vs.* S3_C. The genes in the ovoid circle are the genes involved in anthocyanin degradation. The red parentheses represent the four pathways, the black and red arrows represent the positive regulation and black “T” represent negative regulation. The genes in the red box are the other genes involved in anthocyanin biosynthesis.

The phenylpropanoid and polyketide pathway contained five *Phe-ammonia lyase* (*PAL*), five *C4H*, nine *4CL*, and one *ACC1*. The early biosynthesis contained seven *CHS*, two *CHI*, one *chalcone isomerase-like* (*CHI-L*), two *F3H* and two *F3*′*H*, and the later biosynthesis contained two *DFR*, two *ANS*, eight *UDP-glycosyltransferases* (*UGTs*), one *malnoyl-CoA:anthocyanin 5-O-glucoside-6”-O-malonyltransferase* (*5MAT*), and two *sinapoylglucose:anthocyanin sinapoyltransferase* (*SAT*). Two *TT2*, two *TT19*, and one *ANA10* were involved in anthocyanin transport. Gene *C4H* (*Bo3g024670*) was significantly decreased in S2 *vs.* S1. Certain structural genes had relatively higher Fragments Per Kilobase of transcript per million mapped reads (FPKM) at S1 or S2, including *CHS* (*Bo2g146220*), *F3H* (*Bo8g081770*), *F3*′*H* (*Bo9g174880*) *DFR* (*Bo9g058630*), *ANS* (*Bo1g031790*), and *SAT* (*Bo3g042330*). The FPKM values for most structural genes decreased at S2 (Figure 8). Consistent with the low anthocyanin content at S3_S, the FPKM values for most genes were also very low (Figure 8), and the FPKM values for nearly half of the anthocyanin biosynthesis genes were lower in S3_S than in S3_C. The FPKM values for most of the genes involved in anthocyanin transport (*TT12* and *AHA10*) and modification (*UGT*, *5MAT*, and *A3GlcCouT*) were ˂1. The FPKM values for nearly half the anthocyanin biosynthesis genes were lower in S3_S than in S2. Gene *CHS* (*Bo3g052930*) was significantly decreased in S3_S *vs.* S2 (Figure 9).

**Figure 10:**
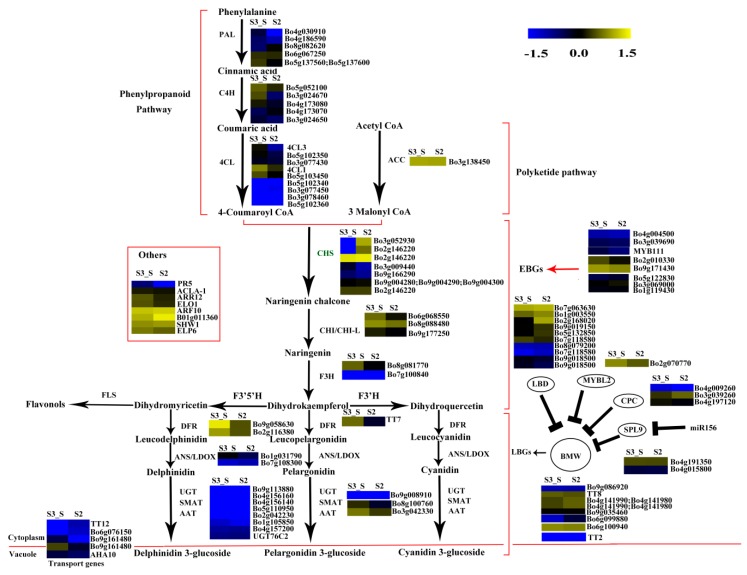
Expression pattern of the genes involved in anthocyanin biosynthesis in S3_S *vs.* S2. Enzyme names in green represent downregulated genes in S3_S *vs.* S2. The red parentheses represent the four pathways, the black and red arrows represent the positive regulation and black “T” represent negative regulation. The genes in the red box are the other genes involved in anthocyanin biosynthesis.

Two of the 34 genes involved in the regulation of anthocyanin biosynthesis were identified as DEGs in pairwise comparisons. *LBD39* (*Bo1g003550*) was upregulated at S2 compared to S1. Almost half of the positive regulatory genes showed a higher expression level at S1 than at S2 (Figure 8). *LBD39* (*Bo7g118580*) and *LBD37* (*Bo2g168020*) were down regulated at S3_S compared to S3_C (Figure 9). One negative regulatory gene, *MYBL2*, exhibited higher level in S3_S than in S3_C. The expression patterns of the anthocyanin biosynthesis genes were consistent with the changes in anthocyanin content at S2 compared to S1, S3_S compared to S3_C, and S3_S compared to S2 (Figure 10).

### 2.8. Expression Patterns of the Genes Involved in Anthocyanin Degrading

The FPKM values of the 36 peroxidase genes were lower than 1. The expression levels of the 16 laccase genes were similar in S2 *vs.* S1, S3_S *vs.* S3_C, and S3_S *vs.* S2. Two peroxidase genes (*Bo4g149600* and *Bo4g187590*) and two β-glucosidase genes (*Bo8g094880* and *Bo4g021730*) were also down regulated in S3_S *vs.* S3_C (Figure 9). The two peroxidase and two β-glucosidase genes were related with the different anthocyanin degrading rates in the leaf center and margin.

### 2.9. DEGs Identified in Two Pairwise Comparisons

In addition to the DEGs mentioned in the previous subsections, other DEGs were found to be related with anthocyanin accumulation. In the pairwise comparison of S2 *vs.* S1, the downregulated *Bo4g098350* was homologous to *AtTIR1*, which is an auxin receptor that mediates auxin-regulated transcriptions (Table S2). Studies also showed that auxin-stimulated flavonoid synthesis was blocked in *TIR1* mutants [19]. A downregulated *DTX* was found in the pairwise comparison of S3_S *vs.* S3_C, and absence of this gene affects flavonoid metabolism [25]. Some TFs were found in these two pairwise comparisons. One *WRKY* TF was downregulated in S2 *vs.* S1. One *MYB*, one *ethylene response factor* (*ERF*), one *MYC-type* (*MYC*) TFs, three *NAC* TFs, and 15 *WRKY* TFs were downregulated and one light harvesting complex photosystem II subunit 6 (*LHCB 6*) was upregulated in S3_S *vs.* S3_C. One photosystem I subunit H2 (*PSAH2*) was upregulated and one photosystem II subunit P-2 (*PSBP-2*) was downregulated in the pairwise comparison of S3_S *vs.* S2. These DEGs may also be involved in color degradation and in bicolor formation at S3 (Tables S3 and S4).

### 2.10. Expression Patterns of the Genes Involved in Chlorophyll Metabolism

**Figure 11:**
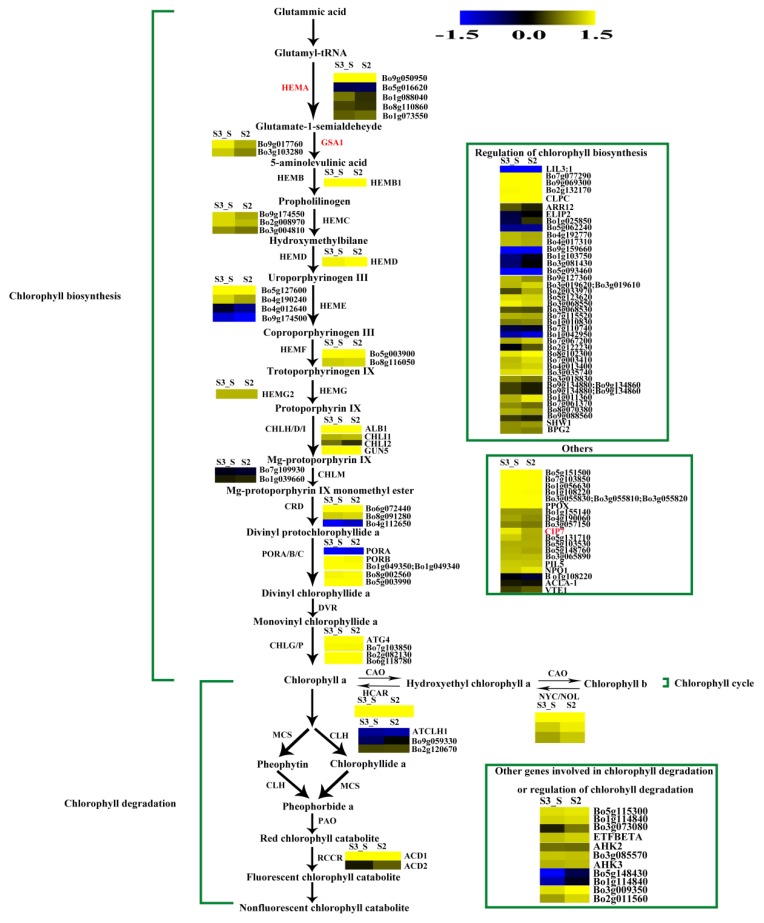
Expression patterns of the genes involved in chlorophyll metabolism in S3_S *vs.* S2. Enzyme names in red represent upregulated genes in S3_S *vs.* S2. The green parentheses represent the three steps in chlorophyll metabolism. The genes in the boxes are the other genes involved in chlorophyll metabolism and the genes involved in regulation of chlorophyll metabolism. The black arrows represent the positive regulation.

The FPKM values for *Bo5g016620*, *Bo4g012640*, *Bo9g174500*, *Bo7g109930*, *Bo4g112650*, *Bo9g115140*, and *Bo1g108220* were ˂1 at all stages. *Bo7g077290*, *Bo9g069300*, *Bo2g132170*, and *Bo3g023990* had relatively high FPKM values (Figure S1). Expression levels changed little for 1/3 of the genes, while those of about another 1/3 of the genes at S2 were higher than at S1. The expression level of this gene in S3_S was over two-fold higher than that in S3_C, but no genes were identified as DEGs in S2 *vs.* S1 and S3_S *vs.* S3_C (Figure S2). Among the 71 genes involved in chlorophyll metabolism, *glutamyl-tRNA reductase* (*HEMA*, *Bo9g050950*), *glutamate-1-semialdehyde 2,1-aminomutase* (*GSA1*, *Bo9g017760* and *Bo3g103280*)*,* and *COP1-interacting protein 7* (*CIP7*, *Bo1g049320*) were DEGs in S3_S *vs.* S2, and these genes were involved in chlorophyll biosynthesis. None of the genes involved in chlorophyll cycle and degradation was found to be DEG in S2 *vs.* S1, S3_S *vs.* S3_C and S3_S *vs.* S2 comparisons (Figure 11).

## 3. Discussion

The bicolored leaves of ornamental kale have attracted extensive attention. However, there has been relatively little research on the formation of these color patterns. In the present study, the pigments, hormones, sugar, and starch contents of bicolor kale leaves were analyzed. RNA sequencing was applied to elucidate the gene expression patterns involved in anthocyanin biosynthesis, anthocyanin degradation, and chlorophyll biosynthesis. The 582.72 million clean reads acquired provided a global view of the transcriptome related with the changes in pigmentation, which occur during plant development. They also helped to identifying the key genes related with bicolor leaf formation.

### 3.1. Anthocyanin and Chlorophyll Content Variations with Plant Development

Leaf color is generally correlated with pigment metabolism. Anthocyanin is generally responsible for the red, blue, pink, and purple leaf colors while chlorophyll is involved in green leaf pigmentation [26,27]. The anthocyanin level slowly decreased with plant development, and at S3 the anthocyanin content in the leaf margin was lower than that in the leaf center. With the increased chlorophyll level in the leaf margin, the leaf finally showed a bicolor leaf phenotype at S3. A previous study showed that the anthocyanin level gradually decreased, and the chlorophyll level slowly increased as the leaf developed in the tea plant [28]. The results of the present study were consistent with that of previous studies. When the anthocyanin/chlorophyll ratio decreased to 2.40, chlorophyll started accumulating. The chlorophyll accumulation was higher when this ratio was lower. Anthocyanin and chlorophyll levels were negatively correlated, and anthocyanin degradation coupled to chlorophyll accumulation led to bicolor leaf formation.

### 3.2. DEGs Involved in Anthocyanin Biosynthesis

In recent years, the genes involved in anthocyanin biosynthesis have been well studied in *Arabidopsis*. Anthocyanin accumulation relies on a series of genes, including structural and regulatory genes. We hypothesized that anthocyanin degrading might be related with a low anthocyanin biosynthesis rate. In the present study, we identified five DEGs in the two pairwise comparisons, including *CHS*, *C4H*, *LBD37*, and two *LBD39*. *C4H* is a key structural gene in the phenylpropanoid pathway, and some *c4h* mutants of *A. thaliana* had lower flavonoid levels than wild type plants. Baek et al. (2008) isolated a full-length cDNA from the *C4H* gene of Korean black raspberry, and found that *C4H* plays an important role in flavanol accumulation during the early stages of fruit ripening [29]. *Bo1g003550* is a homolog of *AtLBD39*, and *LBD39* is a member of the LBD family. Rubin et al. (2009) reported three nitrogen/nitrate-induced members of the LBD gene family, namely, *LBD37*, *LBD38*, and *LBD39* in *A. thaliana*. These genes repress anthocyanin biosynthesis by inhibiting *PAP1* and *PAP2* [12]. Thus, the reduction in anthocyanin content from S1 to S2 might be due to the different expressions of *C4H* and *LBD39*. The downregulated *Bo4g098350* gene *TIR1* was also identified in the S2 *vs.* S1 comparison. *AtTIR1* is an auxin receptor that mediates auxin-regulated transcriptions. Studies have shown that the auxin-stimulated flavonoid synthesis was blocked in *TIR1* mutants [19]. We also detected the auxin contents in S1, S2, S3_C and S3_S and found that the auxin content in S2 was lower than in S1. Thus, and the downregulation of *TIR1* might be due to the decreased auxin content and regulate the low rate of anthocyanin synthesis from S1 to S2. A downregulated *DTX*, which is involved in anthocyanin biosynthesis, was also downregulated in S3_S *vs.* S3_C. And the DEG might be related with pigment changes. Chalcone synthase is a key enzyme of flavonoid biosynthesis that has been identified in many plants. In certain ornamental plants, *CHS* regulates bicoloration in flowers, and some flower patterns are due to post-transcriptional *CHS* silencing. This process occurs in the star and marginal picotee, *Camellia japonica,* and in dahlias [30,31,32]. Therefore, anthocyanin degradation from S2 to S3_S might be due to the expression of DEG.

Recent reports provided insight into the anthocyanin biosynthesis regulated by WRKY TFs. The WRKY TFs mainly regulated the MBW complex and further influenced the anthocyanin biosynthesis [33,34]. The peach NAC TF, BLOOD (*BL*), activates *PpMYB10.1* transcription, and further influences anthocyanin accumulation [15]. In the present study, three NAC TFs and 15 WRKY TFs were denoted as DEGs and were downregulated in S3_S *vs.* S3_C. We also found that the anthocyanin content in the leaf margin differed from that in the leaf center at S3, and that the anthocyanin degrading rate in the leaf margin was faster than that in the leaf center at the same stage. Thus, the DEGs mentioned above may delay anthocyanin degrading in the leaf center and further influence bicolor leaf formation.

### 3.3. DEGs Involved in Anthocyanin Degradation

Most studies on anthocyanin degradation have been conducted in juices and fruits, and few have focused on plants. Anthocyanin often accumulates in young leaves and degrades in mature leaves. This may be due to the fact that anthocyanin can protect leaves from UV light and oxidative damaging, and to the gradual accumulation of chlorophyll in plants [21,35,36], which has been reported in some plants, including *Chrysobalanus icaco*, *Photinia* sp., and *Cocoplum* sp. [21,35,36]. Polyphenol oxidase (PPO), β-glucosidases, peroxidases, and laccases have been reported to be involved in anthocyanin degrading in juices and fruits [22,37,38,39,40,41]. PPO was excluded from the candidate gene pool in plants, but the other three kinds of enzymes might be involved in anthocyanin degrading in plants. β-glucosidases often remove the sugar moieties from anthocyanin, and peroxidases further oxidize the aglycone. Both of these enzymes are located in the vacuole. A laccase is responsible for anthocyanin degrading in litchi fruit pericarp [22] through epicatechin-coupled oxidation, and this enzyme is also located in the vacuole. In the present study, 60 peroxidases, 13 β-glucosidases, and 16 laccases were identified (Table S7). Two peroxidases (*Bo4g149600* and *Bo4g187590*) and two β-glucosidases (*Bo4g021730* and *Bo8g094880*) were downregulated in S3_S *vs.* S3_C. No transcripts of *Bo4g149600* and *Bo4g021730* were detected in S3_S. These four DEGs might be related with the different anthocyanin degrading rates in the leaf center and margin.

### 3.4. DEGs Involved in Chlorophyll Accumulation

The expression patterns of the genes involved in chlorophyll metabolism were also identified in the present study. Four genes were identified as DEGs, including one *HEMA*, two *GSA1* and one *CIP7* in S3_S *vs.* S2. This gene encodes a glutamyl-tRNA reductase protein that catalyzes the nicotinamide adenine dinucleotide phosphate (NADPH)-dependent reduction of Glu-tRNA to glutamate-1-semialdehyde with the release of free tRNA. Kumar et al. (2000) found that *HEMA1* loss-of-function mutants presented patchy to completely yellow color and failed to thrive under normal growth conditions [42]. Schmied et al. (2011) found that *HEMA1* overexpression in etiolated and dark-grown *A. thaliana* and tobacco seedlings can cause protochlorophyllide accumulation [43]. Thus, both *HEMA* and *HEMA1* are involved in the early steps of chlorophyll biosynthesis. *GSA1* is a homologue of glutamate-1-semialdehyde 2,1-aminomtase for catalyzing the conversion of glutamate-1-semialdehyde (GSA) into 5-amino levulinate (ALA). Both, the expression levels of *HEMA* and *GSA1*, were positively regulated by light and led to the formation of ALA, a universal precursor of tetrapyrroles [44]. Yamamoto et al. (1998) found that CIP7 interacts with constitutive photomorphogenic 1 (COP1), which represses photomorphogenesis in darkness. Reduced CIP7 expression decreases anthocyanin and chlorophyll content [45]. In the present study, we identified an upregulated *CIP7* and inferred that its elevated expression might be related with anthocyanin and chlorophyll formation in the green parts of leaves at S3. In addition, the expression levels of the four DEGs were consistent with chlorophyll changes observed during plant development and might lead to chlorophyll accumulation in the leaf margin at S3.

## 4. Materials and Methods 

### 4.1. Plant Materials

Ornamental kale DH lines ‘Y005-7’ were grown in a greenhouse at Shenyang Agriculture University (Shenyang, China). During its ornamental period, this variety displays a bicolor leaf phenotype consisting of red centers and green margins. This phenotype is gradually formed with leaf development, and can be divided into the following stages: stage 1 (S1), leaves entirely red; stage 2 (S2), red pigment appearing in the centers of leaves and leaf margins slightly green; stage 3 (S3), red leaf centers and green leaf margins (Figure 1b). The S1 stage of leaf development occurred from the end of September to mid-October, while the S2 stage occurred from mid-October to the end of October. Next, the S3 stage occurred from November to March of the following year. In order to eliminate the impact of the main vein with higher anthocyanin content, samples at S1. S2 and S3 were collected from whole leaves without the main vein. Samples were immediately frozen in liquid nitrogen and stored at −80 °C before RNA extraction and anthocyanin, chlorophyll, hormone, sugar, and starch content measurements. Three independent biological replicates were used for the above analyses.

### 4.2. Measurement of Chlorophyll and Anthocyanin Contents

Twenty milligrams of freeze-dried leaf tissue were used for pigment extraction. For the chlorophyll measurements, the samples were soaked in 10 mL of a 96% ethanol solution (*v*/*v*) at 25 °C for 24 h, and then whirlpool for 30 s after 12 h in dark culture [46]. A UV spectrophotometer (T6 New Century, Persee, Beijing, China) was used to measure sample absorbances at 649 nm and 665 nm (A_649nm_ and A_665nm_, respectively) based on three replications. The following formulas were used to calculate the pigment content:

Chlorophyll a content = 13.95A_665nm_ − 6.88A_649nm_, (1)

Chlorophyll b content = 24.96A_649nm_ − 7.2A_665nm_. (2)

Anthocyanin content was determined using the method of Rabino and Mancinelli [47]; briefly, 20 mg of freeze-dried leaf tissue was added to 5 mL extracting solution (96% ethanol solution: 1.5 mol/L = 85:15) at 25 °C for 24 h, and then centrifuged for 30 s after 8 h in dark culture. One milliliter solutions were mixed with 2 mL 0.025 M KCl (pH 1.0) and 0.4 M NaAc (pH 4.5). The mixture was cultured at 25 °C for 2 h. Absorbance at 536 nm and 700 nm was measured in triplicate. The following formulas were used to calculate pigment content:

A= (A_536nm_ − A_700nm_) pH 1.0 − (A_536nm_ − A_700nm_) pH 4.5, (3)

(4) $$\mathrm{Anthocyanin}\;\mathrm{content}\;\left(\frac{\mathrm{mg}}{\mathrm{mL}}\right)=\frac{A\times MW\times DF}{e\times 1},$$

*MW* = 449.2 g/mol; *e* = 26900; *DF* = 3.

### 4.3. Measurement of IAA, ABA, GA3, Sugar and Starch Content

Fresh leaf samples (0.3–0.5 g) from different developmental stages and leaf parts were ground in liquid nitrogen, transferred to 3–5 mL isopropyl alcohol/ hydrochloric acid buffer, and kept at 4 °C for 30 min. Six to 10 milliliters of dichloromethane was then added and samples were kept at 4 °C for another 30 min. Liquid samples were centrifuged at 13,000 rpm for 5 min at 4 °C, and the resulting lower organic phase was blow-dried using nitrogen and then dissolved in 400 µL 0.1% formic acid. The solution was filtered through a 0.22-µm membrane and injected in a Qtrap 6500 liquid chromatography (LC)-MS/MS system (ABsciex, Framingham, MA, USA) to determine the IAA, ABA, and GA3 contents using a poroshell 120 SB-C18 column (Agilent Technology, Palo Alto, USA) (2.1 × 150, 2.7 m) set at 30 °C. Phases A and B were methanol/ 0.1% formic acid and water/ 0.1% formic acid, respectively. The gradient was set as: 0–1 min, A at 20%; 1–9 min, an increase to 80%; 9–10 min, A at 80%; 10–10.1 min, A decreased to 20%; 10.1–15 min, A at 20%. The air curtain gas, spray voltage, atomized gas pressure, auxiliary gas pressure, and atomization temperature was 15 psi, 4500 V, 65 psi, 70 psi, and 400 °C, respectively.

Leaf samples (0.1 g) were extracted with 80% ethanol, grind, and then transferred to 10 mL HNO_3_ in boiling water for 10 min. The resulting supernatant was used for measuring total starch content. Sugars were extracted twice using 5–10 mL distilled water in boiling water for 30 min. Three independent biological replicates were used for the above measurement.

### 4.4. Identification of the Genes Involved in Anthocyanin Biosynthesis and Chlorophyll Metabolism

To most comprehensively identified genes involved in anthocyanin biosynthesis and chlorophyll metabolism, the protein sequence of the 40, 33, and 22 genes involved in anthocyanin biosynthesis, chlorophyll metabolism, and regulation of chlorophyll metabolism in *A. thaliana*, respectively, were acquired from TAIR (http://www.arabidopsis.org/). The protein sequences were then aligned with the protein sequences from *Brassica oleracea* (ftp://ftp.ensemblgenomes.org/pub/release-38/plants/genbank/brassica_oleracea/) using the BLASTP (http://plants.ensembl.org/Brassica_oleracea/Tools/Blast?db=core). The threshold was set at E-value ≤ 1 × E^−10^.

### 4.5. Library Construction and Sequencing

A total RNA purification kit (TRK1001; LC Science, Hangzhou, China) was used to extract total RNA from leaves at different stages. The quantity and purity of RNA were determined using Bioanalyzer 2100 and the RNA 6000 Nano LabChip Kit (both Agilent Technologies, Santa Clara, CA, USA). Equal RNA amounts of similar quality were used to create cDNA libraries with the mRNA-Seq sample preparation kit (Illumina, San Diego, CA, USA) following the manufacturer’s protocol. Independent triplicate whole-leaf samples, taken at S1 and S2, and margin (S) and center (C) samples taken at S3 were used to construct 12 cDNA libraries, which were designated S1_1, S1_2, S1_3, S2_1, S2_2, S2_3, S3_S_1, S3_S_2, S3_S_3, S3_C_1, S3_C_2, and S3_C_3. Paired-end sequencing was performed on an Illumina HiSeq 4000 at LC Sciences (Hangzhou, Zhjiang, China). 

Clean reads were obtained by removing the low quality reads (i.e., reads containing sequencing adaptors, sequencing primers, and/or nucleotides with a quality score (Q) <20). After that, clean reads were mapped to the reference genome (ftp://ftp.ensemblgenomes.org/pub/release-38/plants/genbank/brassica_oleracea/) using HISAT (Johns Hopkins University Center for Computational Biology, Baltimore, MD, USA). A maximum of two mismatches and multiple alignments per read (≤20 by default) were allowed.

### 4.6. Transcript Abundance Estimation and Differentially Expressed Genes (DEGs) Testing

StringTie (Johns Hopkins University Center for Computational Biology, Baltimore, Maryland, USA) was used to assemble the mapped reads, and their expression levels were calculated by the FPKM method. Differentially expressed genes were identified by the Ballgown package in R (R Project for Statistical Computing, R Core Team, Johns Hopkins University Center for Computational Biology, Baltimore, Maryland, USA) using log_2_ (fold change) >1 as a threshold and considering *p* < 0.05 as statistically significant. 

### 4.7. GO and KEGG Enrichment Analysis of DEGs

All DEGs were mapped to GO terms and KEGG pathways in the respective databases (GO: http://www.geneontology.org/, KEGG: http://www.kegg.jp/kegg). Significantly enriched GO terms and KEGG pathways were identified using hypergeometric tests and the Bonferroni correction with *p* ≤ 0.05 as a threshold.

### 4.8. GO and KEGG Enrichment Analysis of DEGs

All DEGs were mapped to GO terms and KEGG pathways in the respective databases (GO: http://www.geneontology.org/, KEGG: http://www.kegg.jp/kegg). Significantly enriched GO terms and KEGG pathways were identified using hypergeometric tests and the Bonferroni correction with *p* ≤ 0.05 as a threshold.

### 4.9. qRT-PCR Analysis

In order to verify the reliability of the RNA-seq results, twelve important genes were selected, including five anthocyanin biosynthesis genes (*LBD37*, *C4H*, *DTX35, CHS*, and *LBD39*), two anthocyanin degradation genes (one peroxidase and one β-glucosidase), four chlorophyll metabolism (*HEMA*, two *GSA1*, and *CIP7*) and one auxin receptor (*TIR1*). The *actin* gene (*Bo1g116200*) was used as an internal control. Gene-specific primers were designed with Primer Premier v. 5.0 (PREMIER Biosoft International, Palo Alto, CA, USA) and primer sequences are shown in Table S8. The qRT-PCR was performed in a QuantStudio 6 PCR System (Applied Biosystems, Foster City, CA, USA) using the 2× Ultra SYBR Mixture (CWBio Corp., Beijing, China). All experiments were performed with three independent biological replicates. The relative expression was calculated using the 2^−ΔΔ*C*t^ method [48]. 

### 4.10. Statistical Analyses

Statistical analyses were performed using IBM SPSS statistics 23.0 (https://www.ibm.com/cn-zh/products/spss-statistics). Each value represents the mean ± SD of three independent biological replicates. Significantly different values of qRT-PCR were evaluated using the t-test and *p* ≤ 0.05 as a threshold. Others were evaluated using ANOVA (Tukey test).

## 5. Conclusions

**Figure 12:**
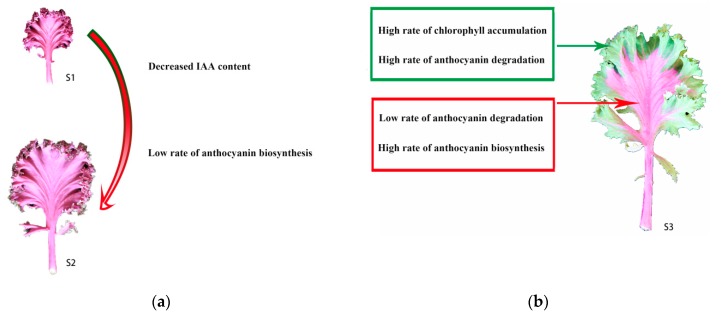
Proposed models for the determination of anthocyanin degrading from S1 to S2 and the bicolor leaf formation at S3. (**a**) Proposed model for the determination of anthocyanin degrading from S1 to S2. The red arrow represent the leaf color change from S1 to S2. (**b**) Proposed models for the bicolor leaf formation at S3. The red box and arrow comprise the biosyntheses occurring in red center leaves and the dark green box and arrow comprises the biosyntheses occurring in green margin leaves.

In the present study, we found that, as the bicolor kale leaf developed, anthocyanin degraded gradually, and chlorophyll accumulated gradually. Candidate genes involved in anthocyanin degradation and anthocyanin biosynthesis were identified by RNA-seq. According to the transcriptome analysis performed here, the anthocyanin degrading in bicolor development is complex. Anthocyanin biosynthesis genes were downregulated from S1 to S2. One auxin receptor, *TIR1*, may block anthocyanin biosynthesis, and might have influenced the anthocyanin degrading from S1 to S2 (Figure 12a). Furthermore, TFs, such as NAC TFs and WRKY TFs were differentially expressed in the leaf margin and leaf center at S3, and might be linked to anthocyanin accumulation at S3. Anthocyanin degrading enzymes like peroxidases and β-glucosidases might play a crucial role in anthocyanin degrading at S3. These DEGs contributed to the bicolor formation at S3 (Figure 12b). One anthocyanin biosynthesis gene was downregulated, and four chlorophyll biosynthesis genes were upregulated during the transition from S2 to S3_S. These five DEGs contributed to chlorophyll accumulation at S3. Therefore, we propose that anthocyanin decreases during bicolor development resulting from reduced anthocyanin biosynthesis and increased anthocyanin degradation, while chlorophyll accumulation during bicolor development is a result of increased chlorophyll biosynthesis.

## Supplementary Materials

Supplementary materials can be found at http://www.mdpi.com/1422-0067/20/3/603/s1. **Figure S1**: Expression patterns of the genes involved in chlorophyll biosynthesis in S2 *vs.* S1. The green parentheses represent the three steps in chlorophyll metabolism. The genes in the boxes are the other genes involved in chlorophyll metabolism and the genes involved in regulation of chlorophyll metabolism. The black arrows represent the positive positive regulation. **Figure S2**: Expression patterns of the genes involved in chlorophyll biosynthesis in S3_S *vs.* S3_C. The green parentheses represent the three steps in chlorophyll metabolism. The genes in the boxes are the other genes involved in chlorophyll metabolism and the genes involved in regulation of chlorophyll metabolism. The black arrows represent the positive positive regulation. **Table S1**: Statistics of RNA-Seq data. **Table S2**: Summary of the 201 DEGs in S2 *vs.* S1. **Table S3**: Summary of the 543 DEGs in S3_S *vs.* S3_C. **Table S4**: Summary of the 391 DEGs in S3_S *vs.* S2. **Table S5**: Genes involved in anthocyanin biosynthesis. **Table S6**: Genes involved in chlorophyll metabolism. **Table S7**: Genes involved in anthocyanin degrading. **Table S8**: Primers used to validate the expression patterns.

## Abbreviations

DH	double haploid
IAA	indole-3-acetic acid
ABA	abscisic acid
GA3	gibberellin 3
DEGs	differentially expressed genes
C4H	cinnamate-4-hydroxylase
TIR1	transport inhibitor response 1
LBD	lateral organ boundaries domain
BGLU	β-glucosidase
DTX35	detoxifying efflux carrier 35
NAC	no apical meristem
WRKY	WRKY DNA-binding protein
TFs	transcription factors
CHS	Chalcone synthase
CHI	chalcone isomerase
CoA	coenzyme A
F3H	flavanone 3-hydroxylase
F3′H	flavonoid 3′-hydroxylase
DFR	Dihydroflavonol reductase
LDOX/ANS	leucoanthocyanidin dioxygenase/anthocyanidin synthase
ANR	anthocyanidin reductase
GST	glutathione-S-transferase
ABC	ATP-binding cassette
ATP	adenosine triphosphate
MATE	toxic compound extrusion
MYB	myeloblastosis
HYH	HY5 homolog
HY5	elongated hypocptyl 5
MYBL2	MYB-like 2
CPC	caprice
SLP9	squamosa promoter binding protein-like 9
PAP	production of the anthocyanin pigment
CK	cytokinin
UV	ultraviolet
ADE/LAC	laccase
ANOVA	the analysis of variance
BLASTP	the protein query basic local alignment search tool
EC	Enzyme
PAL	Phe-ammonia lyase
CHI-L	chalcone isomerase-like
UGTs	UDP-glycosyltransferases
MAT	malnoyl-CoA:anthocyanin 5-O-glucoside-6”-O-malonyltransferase
SAT	sinapoylglucose:anthocyanin sinapoyltransferase
FPKM	Fragments Per Kilobase of transcript per million mapped reads
ERF	ethylene response factor
MYC	MYC-type
HEMA	glutamyl-tRNA reductase
GSA1	glutamate-1-semialdehyde 2,1-aminomutase
CIP7	COP1-interacting protein 7
NADPH	nicotinamide adenine dinucleotide phosphate
GSA	glutamate-1-semialdehyde
ALA	5-amino levulinate
COP1	constitutive photomorphogenic 1
LHCB 6	light harvesting complex photosystem II subunit 6
PPO	Polyphenol oxidase
GO	Gene Ontology
KEGG	Kyoto Encyclopedia of Genes and Genomes
