# Supplementary figures and images for: Anthocyanin Degrading and Chlorophyll Accumulation Lead to the Formation of Bicolor Leaf in Ornamental Kale
Source: Int J Mol Sci. 2019 Jan 30;20(3):603. doi: 10.3390/ijms20030603 (PMC6387137; doi:10.3390/ijms20030603)

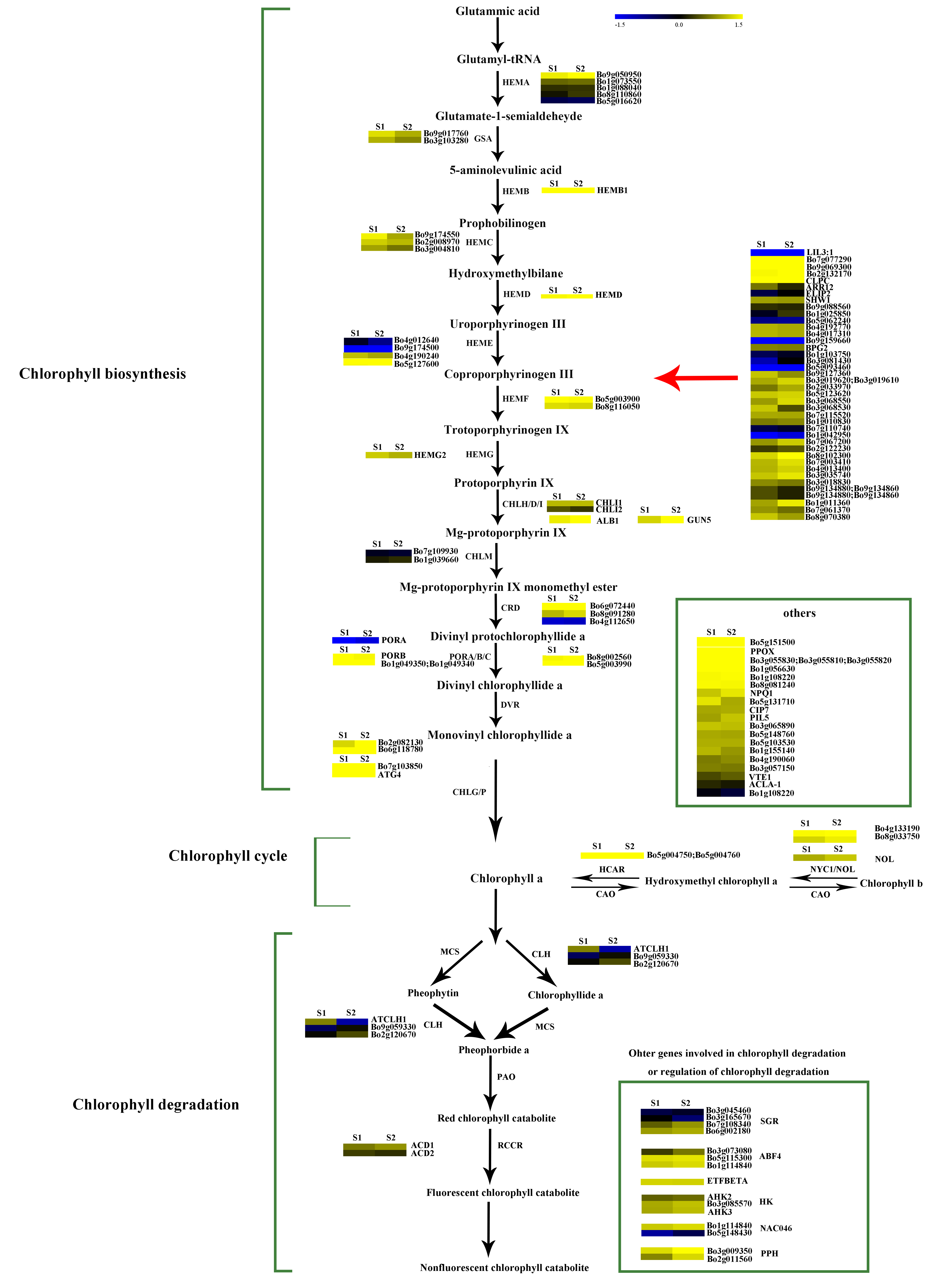

Supplement: Supplementary file 1 [file ijms-20-00603-s001.zip › Supplementary/Figure S1.png]

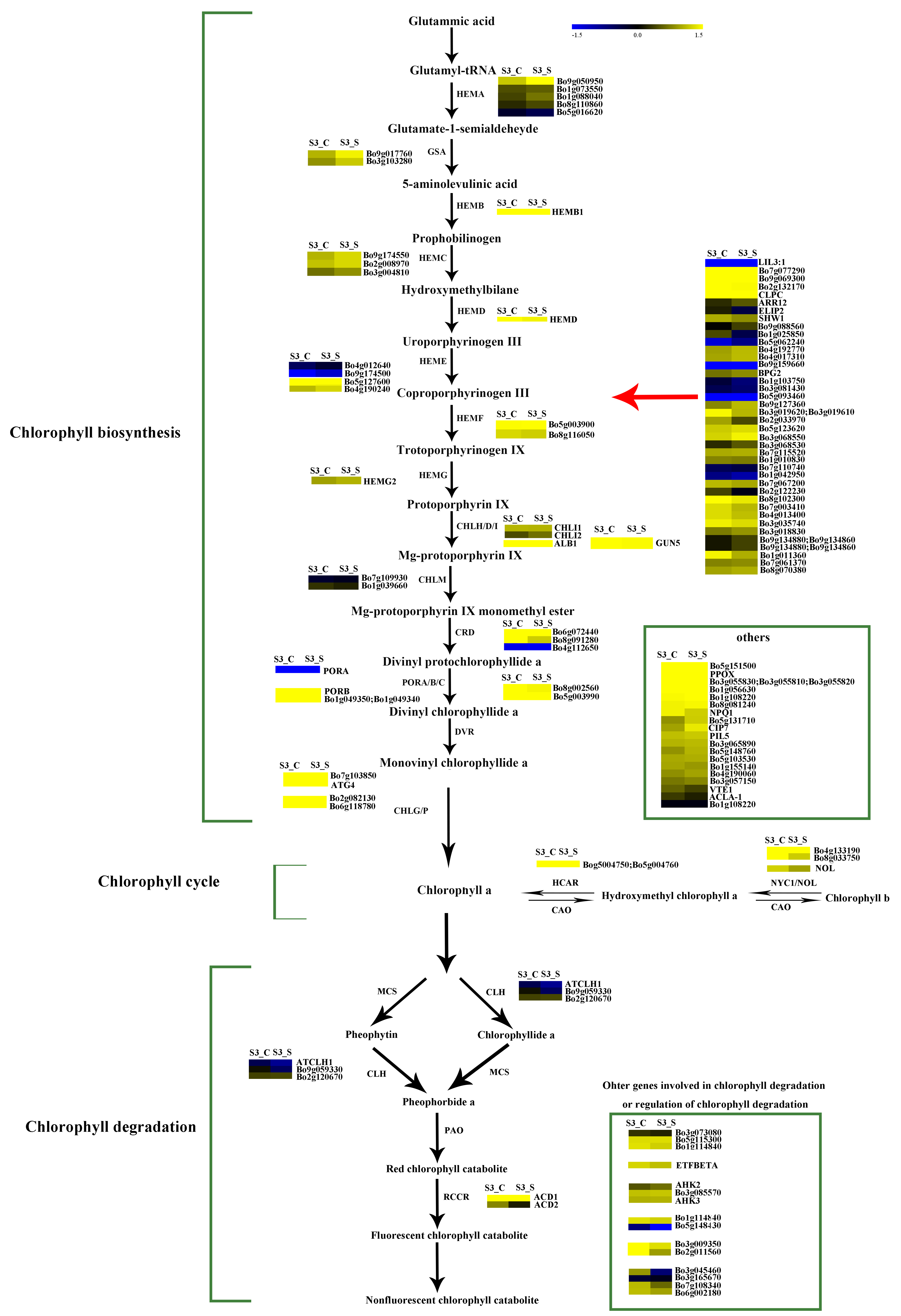

Supplement: Supplementary file 1 [file ijms-20-00603-s001.zip › Supplementary/Figure S2.png]
